# Mobile Phone Use in a Car-Following Situation: Impact on Time Headway and Effectiveness of Driver’s Rear-End Risk Compensation Behavior via a Driving Simulator Study

**DOI:** 10.3390/ijerph17041328

**Published:** 2020-02-19

**Authors:** Yunxing Chen, Rui Fu, Qingjin Xu, Wei Yuan

**Affiliations:** 1 School of Automobile, Chang’an University, Xi’an 710064, China; chenyunxing@chd.edu.cn (Y.C.); qingjinxu@outlook.com (Q.X.); yuanwei@chd.edu.cn (W.Y.); 2 School of mechanical engineering, Hubei University of Arts and Science, Xiangyang 441053, China

**Keywords:** distraction, speech-based texting, handheld texting, time headway, rear-end accident probability, compensation behavior

## Abstract

Mobile phone use while driving has become one of the leading causes of traffic accidents and poses a significant threat to public health. This study investigated the impact of speech-based texting and handheld texting (two difficulty levels in each task) on car-following performance in terms of time headway and collision avoidance capability; and further examined the relationship between time headway increase strategy and the corresponding accident frequency. Fifty-three participants completed the car-following experiment in a driving simulator. A Generalized Estimating Equation method was applied to develop the linear regression model for time headway and the binary logistic regression model for accident probability. The results of the model for time headway indicated that drivers adopted compensation behavior to offset the increased workload by increasing their time headway by 0.41 and 0.59 s while conducting speech-based texting and handheld texting, respectively. The model results for the rear-end accident probability showed that the accident probability increased by 2.34 and 3.56 times, respectively, during the use of speech-based texting and handheld texting tasks. Additionally, the greater the deceleration of the lead vehicle, the higher the probability of a rear-end accident. Further, the relationship between time headway increase patterns and the corresponding accident frequencies showed that all drivers’ compensation behaviors were different, and only a few drivers increased their time headway by 60% or more, which could completely offset the increased accident risk associated with mobile phone distraction. The findings provide a theoretical reference for the formulation of traffic regulations related to mobile phone use, driver safety education programs, and road safety public awareness campaigns. Moreover, the developed accident risk models may contribute to the development of a driving safety warning system.

## 1. Introduction

Mobile phone use while driving is a very common phenomenon, and it has become one of the main factors in the occurrence of traffic accidents all across the world [1,2,3]. Although many countries prohibit the use of mobile phones during driving, this phenomenon is still very widespread. For instance, previous studies in China have shown that 84.1% of drivers (*n* = 414) reported talking on a phone at least once a week [4]. In the United States (*n* = 3265), a study based on roadside observations found that 48% of all distracted drivers engaged in using mobile phones while driving [5]. In European countries such as Spain, it has been reported that the proportion of drivers for text messaging, having a handheld or hands-free conversation is 43.7%, 32.2%, and 25.4%, respectively [6]. A cross-sectional study in Australia indicated that nearly 50% of drivers (*n* = 484) engaged in mobile phone conversations or texting/browsing during driving on a typical day [7]. According to the World Health Organization (WHO), the proportion of people who engage in texting while driving around the world is still very high: 27%, 45%, and 16.67% in the United States, the UK, and Australia, respectively [8]. In a survey taken in New Zealand (*n* = 1057), Lambert and Regan [9] found that more than 50% of drivers admitted to sending or reading 1–5 text messages per week during driving. The widespread use of mobile phones while driving has led to an increased risk of vehicular accidents. For example, the NHTSA (National Highway Traffic Safety Administration, 2019) indicated that in the United States in 2017, 401 fatal crashes (14% of all fatal distraction-affected crashes) were reported to have involved the use of mobile phones [2]. The WHO research suggested that drivers who use mobile phones were about four times more likely to be involved in an accident than those without mobile phones [3].

Mobile phone distracted driving can impair a driver’s car-following performance. For example, mobile phone distraction driving reduces driving speed [10], increases following distance [11], and increases time headway [12]. However, research shows that these performance impairments may not be completely harmful; in some cases, drivers may take conscious or unconscious compensation behavior to compensate for the increased workload associated with mobile phone use [10,13]. Many studies have shown that the types of mobile phone use have a great impact on a driver’s compensation strategies. The types of mobile phones that have been studied by numerous scholars in terms of speed reduction include the following: handheld phone conversations [10,11,14,15,16]; hands-free phone conversations [12,14,16,17,18]; handheld texting [10,11,15,19,20,21,22]; and speech-based texting [23]. Increasing following distance was observed for all types of mobile phone use: handheld phone [11,12,17]; hands-free phone [12,18]; handheld texting [11,24]; and speech-based texting [25]. Mobile phone use related to increased time headway that has been investigated by scholars includes handheld phone conditions [12]; hands-free phone conditions [12]; handheld texting conditions [26]; and speech-based texting conditions [27]. In summary, many scholars have studied the effects of mobile phone use while driving on car-following performance (speed, following distance, time headway). Most scholars’ research on drivers’ various compensation behaviors has focused on reducing speed and increasing following distance. Whereas, very little research has focused on considering the speed and the following distance together, and using time headway as an indicator to study the compensation behavior in the car-following environment. Li et al. [28] indicated that reducing speed or increasing following distance as a compensatory strategy is not sufficient in a car-following situation, and that drivers should negotiate their speed and following distance simultaneously. To maintain driving safety, drivers compensate for the increased accident risk caused by using mobile phones by exercising only one type of compensation behavior (reducing speed, increasing following distance, or increasing time headway). Some scholars have studied the impact of mobile phone distraction on the time headway, but very few scholars have studied whether time headway compensation strategies can completely offset the increased accident risk caused by mobile phone distraction, especially in the case of sudden braking of the lead vehicle.

Mobile phone distraction tasks can impair drivers’ perception of dangerous events and increase their accident risk [13,29]. Choudhary and Velaga [10] showed that the accident probability increased by three and four times, respectively, when drivers engaged in handheld phone conversations or texting. Many scholars have indicated that the increased accident risk caused by handheld texting is even higher than that caused by handheld mobile phone conversations [24,30]. The results of the SHRP2 Natural Driving Data Study conducted by the University of Virginia showed that the accident rate for drivers who were texting while driving increased by 6.46% [31]. Similarly, many research results show that the accident risk caused by writing or reading text messages is significantly increased [21,32,33]. Most studies have focused on the impact of engaging in mobile phone conversations and texting on accident risk, but few scholars have studied the effects of speech-based texting on rear-end accident risk. Zhang et al. [34] demonstrated that speech-based texting while driving is more dangerous than hands-free mobile phone conversations. Some studies have opined that speech-based texting during driving is safer than handheld texting [35,36]. In addition, situational urgency has an important impact on accident risk, and this factor has been measured using two types of indicators: initial time headway (time headway at lead vehicle brake onset) and lead vehicle deceleration [37,38]. Car-following is a common and complex driving behavior. Drivers have to continuously pay attention to the driving state of the lead vehicle (LV). The initial time headway could be more influential than the mean time headway in terms of accident risk in car-following situations. As the initial time headway can better reflect the level of situational urgency, it has a direct impact on the rear-end accident risk [29,38,39]. However, the effects of mobile phone distracted driving (i.e., speech-based texting and handheld texting) on rear-end accident risk under varying levels of situational urgency has not been very well studied.

Most drivers would take conscious or unconscious self-regulatory behaviors when using mobile phones to avoid the increased risk of accidents related to mobile phone distraction [14,40,41]. Oviedo-Trespalacios et al. [14] introduced a behavioral adaptation theory related to this concept. The compensation strategy adopted by the driver during mobile phone distracted driving comprises three types: strategic control, tactical control, and operation control. Strategic control is not to perform secondary tasks while driving (e.g., the driver may turn off any mobile phones before driving), while tactical control is the management of time spent on secondary tasks (e.g., adjusting the time of use of the mobile phone), and operational control refers to adjusting the driving tasks when the driver is engaged in secondary tasks (e.g., increasing time headway or reducing speed) [14]. Numerous scholars have used this theory to study the mechanism of behavioral adaptation of mobile phone distracted drivers [14,42,43]. The current study is limited to operational control, especially the time headway adaptation behavior of mobile phone distracted drivers. Up to now, many studies have shown that a driver’s speed compensation behavior may not always completely offset the increased risk of accidents related to mobile phone distraction, especially in dangerous emergencies [10,13,44,45]. However, research on the effectiveness of the time headway compensation behavior during mobile phone distracted driving is not known from the existing literature.

The above-mentioned research limitations can be summarized as follows. Little research has been conducted that attempts to quantify the effects of speech-based texting and handheld texting (two difficulty levels in each task) while driving on the time headway and the probability of rear-end accidents considering different levels of situational urgency. In addition, the relationship between a driver’s time headway increase strategy and rear-end accident risk when driving while affected by mobile phone distraction (i.e., engaging in speech-based texting and handheld texting) has not been well studied. Further, using the actual experimental data statistics to study the effectiveness of time headway compensation behavior during mobile phone distracted driving in the car-following situation has not been found from the existing literature. To date, numerous scholars’ research on the influence of mobile phone distraction on driving performance mainly concentrates on hand-held conversations, hands-free conversations, and texting, but few scholars have focused on speech-based texting. The impact of speech-based texting on driving safety has been studied in developed countries such as the United Kingdom and the United States, most of the communication information in these countries is in the English language, and speech-based texting is sent and received through the Siri or Vlingo software [36]. In China, however, it is extremely common for drivers to receive and send speech-based texting while driving via WeChat software. WeChat’s operational interface, language input system, speech-based texting length, and Chinese usage habits are very different from those in other countries. Simply put, there are few studies on the effect of a driver’s mobile phone usage habits on his or her time headway and rear-end accident probability. Finally, most studies that do exist on these topics have used variance analysis to qualitatively analyze the impact of distracted driving on driving performance [23,24,27,46]. However, few statistical models have been established to quantitatively analyze the impact of mobile phone distraction on the time headway and rear-end accident probability of drivers who use their phones while driving.

To address the above-mentioned research gaps, this study investigates the effects of speech-based texting and handheld texting (each with two difficulty levels) on drivers’ time headway and rear-end accident probability under the different time headway and LV decelerations of 3, 5, and 8 m/s^2^. Moreover, in order to investigate the effectiveness of the time headway compensation behavior, the relationship of time headway increase strategies during mobile phone use and the corresponding rear-end accident frequencies is also studied based on actual experimental data statistics. Some specific aspects of this study are summarized as follows: (1) rear-end conflict events in the Chinese urban road environment are designed in a driving simulator that examines drivers’ situation awareness. (2) Both speech-based texting and handheld texting include two levels of difficulty. (3) The Generalized Estimating Equation (GEE) model is used to test the effects of mobile phone use on time headway and rear-end accident probability. (4) All factors (driver demographics, driving history, mobile phone use habits, and different distraction conditions) are considered to analyze car-following performance. (5) Finally, the present study attempts to quantify the extent to which time headway increase strategies can offset the increased rear-end accident risk related to the use of mobile phones while driving. Furthermore, the effectiveness of driver’s rear-end risk compensation behavior is studied through the statistics of actual experimental data.

## 2. Methodology

### 2.1. Participants

A total of 54 drivers with valid driver’s licenses participated in this experiment. One participant failed to complete the experiment due to motion sickness. Thus, 53 participants were selected for the study. The study protocol was approved by the Ethics Committee of Chang’an University. A questionnaire was used to collect basic information about each participant, including driver demographics, driving history, and mobile phone use habits. This basic information was used to create explanatory variables for establishing performance models. Descriptive statistics of the participants obtained from the questionnaire are presented in Table 1. The participants were between 22 and 34 years old (M = 25.25, SD = 3.08; 37 males and 16 females). The average driving experience of all participants was 3.02 (SD = 2.27) years. About 79.25% drove less than 5000 km; 9.43% drove between 5000 and 10,000 km; and the remaining 11.32% drove more than 10,000 km. About 96.23% of the participants had had no traffic accidents, and the percentage of those with only one traffic accident and of those with more than one traffic accident was 1.89% for the past three years. None of the participants had had a traffic accident due to mobile phone use. The statistics of the mobile phone use habits of all the participants showed that most of them preferred to receive and send speech-based texting via WeChat while driving, compared to engaging in handheld texting. About 41.51% of the participants sometimes used speech-based texting via WeChat, and 18.87% of them frequently used it. About 33.96% of the participants sometimes used handheld texting via WeChat, and only 1.89% often used handheld texting.

### 2.2. Apparatus

**Figure 1:**
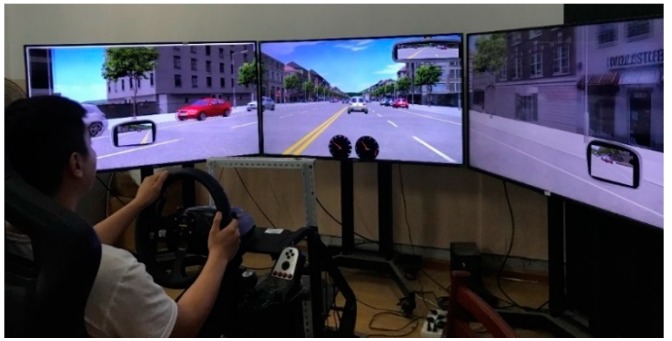
The driving simulator.

The experiment was carried out based on a fixed-base driving simulator (see Figure 1). The display system consisted of three 55-inch HD screens with a horizontal view of 120°. The driving simulator was an automatic transmission vehicle, and the driver needed to operate only the steering wheel and the accelerator and brake pedals. Additionally, rear and side-view mirrors, speedometers, and tachometers were displayed on the screen. Sound systems were used to simulate engine and road noise. The driving simulator computer system recorded the vehicle operation data, including the following distance and the subject vehicle (SV) speed, and the data sampling frequency was 60 Hz.

### 2.3. Simulation Scenarios

The simulation scenarios used in the study included a two-way four-lane road of 3 km (each lane was 3.5 m in width), with a posted speed limit of 60 km/h and two non-motor vehicle lanes. The road environment was created with the characteristics of urban roads in China. In order to eliminate the interference of other vehicles moving in the driving direction, only two vehicles (i.e., the SV and the LV) drove in the inner lane. The speed of the lead vehicle was 50 km/h, and there was a certain traffic flow in the opposite lane. Each participant had to drive in five different driving conditions: normal (no phone use), simple speech-based texting, complex speech-based texting with difficulty level, simple handheld texting, and complex handheld texting with difficulty level.

Each participant needed to drive in five different car-following conditions on a 3 km long straight road. Every time the SV started from the starting point and moved to a certain position, the LV was triggered to accelerate from 0 to 50 km/h with an acceleration of 1 m/s^2^, and it then drove at a constant speed of 50 km/h. Each driving condition required the participants to follow the lead vehicle according to their own driving habits. To test the driver’s ability to perceive the rear-end conflict event, the LV was programmed to make three unexpected full stops with brake lights on in each driving condition, and the three decelerations of the LV were 3, 5, and 8 m/s^2^ [38]. In order to avoid a rear-end collision, the participants performed braking behavior according to their typical driving habits. When the operating speed of the SV reached the minimum value or 0, the rear-end collision avoidance behavior ended. The LV then again accelerated from 0 to 50 km/h with an acceleration of 1 m/s^2^ and then drove at a constant speed. Then, the SV continued to follow the LV. Meanwhile, the experimenter recorded the data of the rear-end collision that occurred during each experiment. In total, 85 rear-end accidents happened during the sudden braking event. Among them, the number of accidents of normal (no phone use), simple speech-based texting, complex speech-based texting, simple handheld texting, and complex handheld texting are 11, 17, 17, 18, 22, respectively.

### 2.4. Secondary Tasks

The secondary tasks that characterize distracted driving included two types of speech-based texting and handheld texting. They were designed to include two levels of difficulty: simple and complex. The simple tasks were replaced by single-digit addition and subtraction operations within 10, and complex tasks were double-digit addition and subtraction operations within 100 [28,34,47]. Messages were sent and received between the experimenter and the participant via the WeChat software during the secondary tasks. When the participant received speech-based texting, he or she was asked to reply only by means of speech-based texting; the participant could not reply by handheld texting. The same restrictions were applied to handheld texting; if the participant received a handheld texting, he or she had to reply using handheld texting and not speech-based texting.

### 2.5. Procedure

After the participants arrived for the experiment, the experimenter introduced the experimental requirements to each participant and asked them to read and sign the informed consent form. Then they were asked to fill out a questionnaire. The details of the questionnaire are shown in Table 1. Then, the experimenter introduced the experimental requirements to the participants. Before starting the formal experiment, each participant took about 5–10 min to perform driving operations in the driving simulator, including acceleration, deceleration, and turning, until they became fully familiar with the simulator. 

During the formal experiment, each participant needed to perform five driving tasks: one non-distracted driving session and four distracted driving sessions with different mobile phone use tasks (simple and complex speech-based texting, simple and complex handheld texting). The experimenter sat in a room separated from the driving simulator and sent WeChat messages to the participants, and the participants were requested to reply to the WeChat messages as soon as possible after receiving them. Then, the experimenter immediately sent the next message, and the communication process continued until the driving task was over. To avoid the influence on the experimental results of driver fatigue caused by long driving time, the participants rested for at least 5 min between every two tests. Meanwhile, in order to avoid any influence on the results from the learning effect, the order of driving tasks among all the participants, and the deceleration behavior of the LV, were random. Further, the participants were required to obey traffic rules and speed limits and to complete the driving experiment using their typical, true driving habits. If the participant felt discomfort, such as motion sickness, during the test, the experiment could be ended at any time. The driving simulation time of each participant was about 30 min.

### 2.6. Analysis

#### 2.6.1. Dependent and Independent Variables

The present study analyzed the impact of the different difficulty levels of mobile phone tasks on time headway and rear-end accident risk under different decelerations of the LV in a car-following situation. The time headway at LV brake onset and the rear-end accident probability were taken as dependent variables to establish the models. Driving conditions, driver demographics, driving history, and mobile phone use habits were considered independent variables in the model. Driving conditions included these factors: no phone use, speech-based texting, difficulty in speech-based texting, handheld texting, and difficulty in handheld texting. Here, the variable “Speech-based texting” represented the appearance of speech-based texting tasks (e.g., whether the driver was using speech-based texting through the WeChat software). However, the variable “difficulty in speech-based texting” represented the appearance of complexity in the speech-based texting tasks (e.g., whether it was a complex speech-based texting task or a simple speech-based texting task). Similarly, the variable “handheld texting” represented the appearance of handheld texting, and the variable “difficulty in handheld texting” represented the appearance of complexity in the handheld texting tasks. The statistical details of other independent variables (driver demographics, driving history, and mobile phone use habits) are shown in Table 1.

#### 2.6.2. Statistical Approach

In this paper, the experimental data was gathered from multiple participants, with multiple observations for each participant. Therefore, our data may contain correlations among multiple observations due to repeated observations of the same driver. To solve this problem, the Generalized Estimating Equation (GEE) method was used to build the dependent variable model in Stata Software, which can be used for continuous variables and categorical variables [12,48]. The GEE model for time headway was developed using an identity link function. Whereas, a logit link function was used for the rear-end collision risk model, because the dependent variable was a binary variable (1 if an accident occurred and 0 otherwise). Although GEE can accommodate several correlation structures, this study used an exchangeable correlation structure that assumed a constant correlation coefficient between repeated observations from the same participant [12,48,49].

## 3. Results

### 3.1. Modelling Time Headway

**Figure 2:**
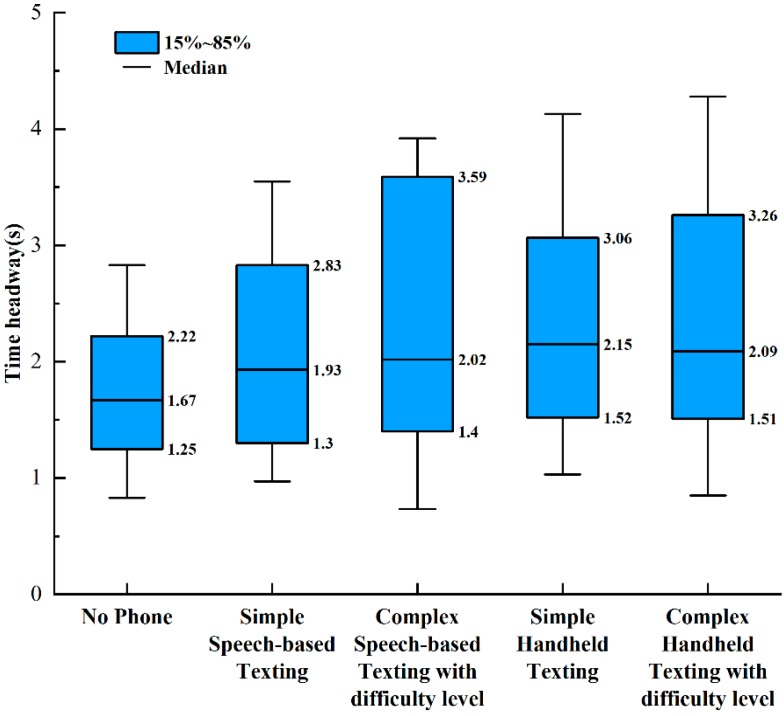
Boxplots of initial time headway in different driving conditions.

Each driver followed the LV at a comfortable distance and speed in car-following situations. The drivers’ following distance and speed were affected by many factors, such as driver demographics, driving characteristics, road driving environment, and mobile phone secondary tasks. Hence, each driver’s following distance and speed were different. The present paper took the time headway as the driver’s risk compensation behavior parameter, which had a practical significance, because both car-following distance and speed were considered. The boxplots shown in Figure 2 demonstrate the initial time headway (time headway at LV brake onset) of the 53 participants in different driving conditions according to the minimum, maximum, 85th, 50th (median), and 15th percentile values of time headway. As can be seen from Figure 2, compared with non-distracted driving, the driver’s risk compensation behavior (increasing the time headway) can be observed under the distracted driving conditions (speech-based texting and handheld texting). Further, the driver’s time headway selection during non-distracted driving was relatively concentrated, which indicates that the choices of driver’s time headway were similar. However, the drivers chose time headway values that spanned a considerable range during the periods of distracted driving conditions, indicating that there were different levels of risk compensation behavior among the drivers.

To quantify the impact of mobile phone use and other factors on time headway, the Generalized Estimating Equation (with identity link function) model was used. Two separate models were developed for two different distracted driving conditions (speech-based texting and handheld texting). All variables in Table 1 and all the driving conditions were taken as independent variables, and time headway (in seconds) was taken as a dependent variable. In the process of selecting independent variables, the multicollinearity of the variables was carefully examined to develop the models. The final model results are presented in Table 2. Table 2 shows the estimated coefficients, SE (standard error), z value, and *p* > |z| for all significant factors. A chi-square test was used to verify the goodness of fit for the two models. The results of the time headway models for speech-based texting and handheld texting show that the values of Prob > chi2 were 0.0002 and 0.0000, respectively. Thus, it can be considered that the two developed models had significant goodness of fit, and the fitting results of the models were acceptable.

The results shown in Table 2 indicate that both speech-based texting and handheld texting had significant effects on the time headway. Compared with non-distracted driving, speech-based texting caused time headway to increase by 0.41 s, while handheld texting led to a greater increase in time headway (0.59 s). Interestingly, the effect of the difficulty level of the secondary task (difficulty in speech-based and handheld texting) on the time headway proved to be insignificant. However, the results of the two models also showed that the driver’s gender and driving history had no significant effect on time headway. Interestingly, both models also indicated that mobile phone use habits had no significant effect on the time headway, and further suggested that frequent and infrequent use of mobile phones caused the same level of risk perception.

### 3.2. Modelling Rear-End Accident Probability

In a car-following situation, the influence of different driving conditions (no phone use, speech-based texting, difficulty in speech-based texting, handheld texting, and difficulty in handheld texting) on rear-end accident risk was studied during different time headway choices and with the LV decelerations of 3, 5, and 8 m/s^2^. In order to quantify the impact of mobile phone distraction and other factors (time headway, driver demographics, driving history, and mobile phone use habits) on rear-end accident risk, two accident risk models were developed (speech-based texting and handheld texting) using the GEE method. The rear-end accident probability was taken as the dependent variable and was expressed as a binary variable: 1 if the rear-end accident occurred due to the LV sudden braking, otherwise 0. The link function is a logit function, and the calculation formula of rear-end accident probability is as follows:

(1) $$p=\frac{e^{X_{ij}\beta }}{1+e^{X_{ij}\beta }}$$

where p is rear-end accident probability; $X_{ij}$ is the matrix of independent variables representing the variables shown in Table 1 (i.e., driver demographics, driving history, and mobile phone use habits) and the distracted driving conditions (presence of speech-based texting and handheld texting); and β is a coefficient estimate matrix of the corresponding variables. Two different binary logic models were developed for speech-based texting and handheld texting that considered all of the explanatory variables presented in Table 1, the initial time headway, the LV deceleration, and all the different distraction conditions. The effect of the initial time headway on the rear-end accident probability was studied by maintaining the initial time headway as a continuous variable in the two models. The LV deceleration was a categorical variable as an independent variable in this study. The results of the two models are shown in Table 3. Table 3 includes parameter variables, parameter estimation coefficients, SE (Standard Error), z value, *p* > |z|, and OR (odds ratio). A chi-square test was used to check the goodness of fit for both models. The two models for speech-based texting and handheld texting indicated that the values of Prob > chi2 were 0.0000. Therefore, the results of the two models were reasonable.

**Figure 3:**
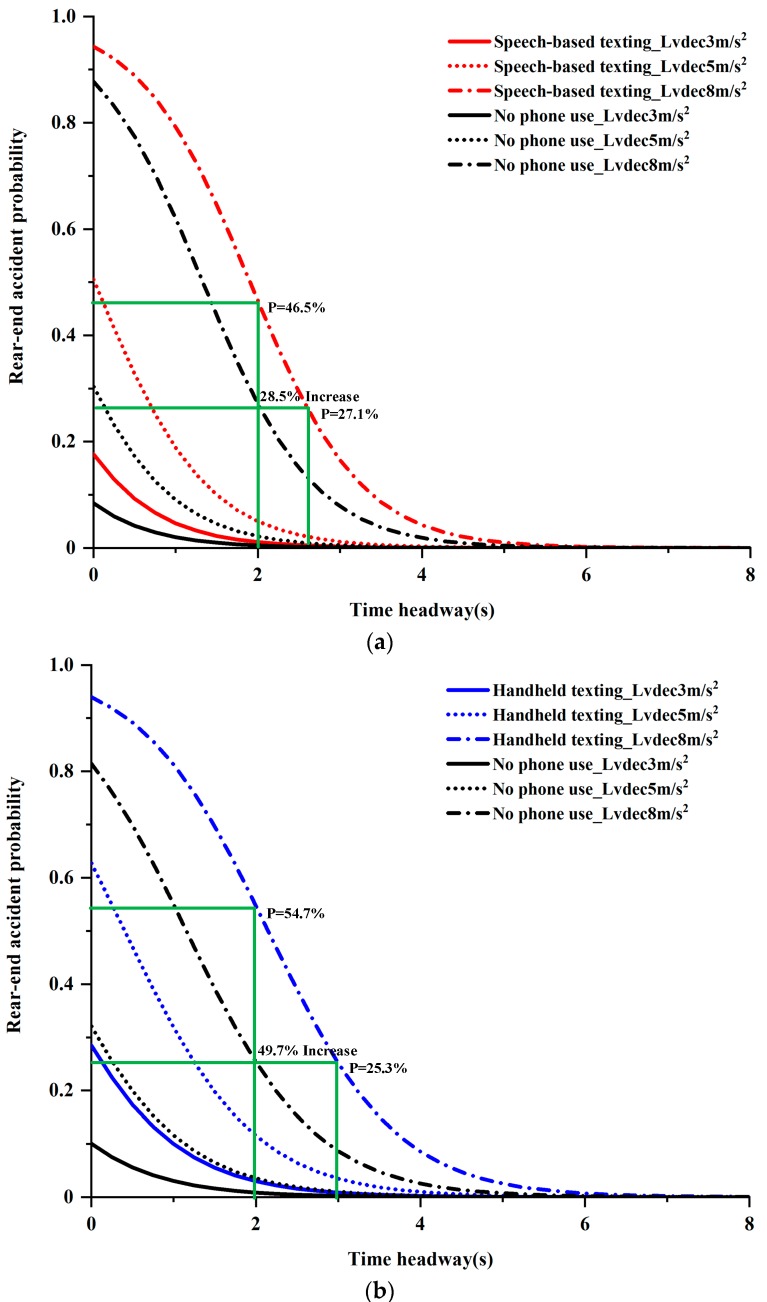
Comparison of rear-end accident probabilities for non-distracted and distracted driving conditions with different time headways and LV deceleration rates. (**a**) Comparison of rear-end accident probability between speech-based texting and no phone use; (**b**) comparison of rear-end accident probability between handheld texting and no phone use.

The odds ratio (OR) shown in Table 3 demonstrates that, compared to non-distraction driving, the presence of speech-based texting and handheld texting increased the rear-end accident probability by 2.34 and 3.56 times, respectively, while the difficulty level of the secondary tasks had no significant effect on rear-end accident probability. Simultaneously, the results of the rear-end risk model shown in Table 3 indicate that the deceleration of the LV had a significant impact on the accident probability. The model of speech-based texting shown in Table 3 demonstrates that, compared with an LV deceleration rate of 8 m/s^2^, the rear-end accident probability at an LV deceleration rate of 3 m/s^2^ was reduced by 99%, and at an LV deceleration rate of 5 m/s^2^, it was reduced by 94%. Similarly, the model of handheld texting presented in Table 3 indicates that, compared to an LV deceleration rate of 8 m/s^2^, the rear-end accident probability with LV deceleration rates of 3 and 5 m/s^2^ reduced by 97% and 89%, respectively. The above evidence shows that the lower the LV deceleration rate, the lower the accident risk (as can be seen from Figure 3). Similar to previous studies, the driver’s gender, and mobile phone use habits had no significant impact on rear-end accident risk [10,21]. The model also shows that driving history was not significant.

As can be gleaned from the discussion in Section 3.1, to compensate for the increased rear-end accident risk caused by mobile phone distraction tasks, the drivers adopted compensation behaviors and increased their time headway. Results of the models of speech-based texting and handheld texting shown in Table 3 demonstrate that a 1 s increment in initial time headway reduced the accident risk by 77% (speech-based texting) and 72% (handheld texting). To better understand the quantitative relationship between time headway, LV deceleration, different driving conditions, and rear-end accident probability, the two rear-end risk models based on Equation (1) and the estimated coefficients of the parameters shown in Table 3 can be expressed as follows:

Rear-end accident risk model of speech-based texting:

(2) $$p=\frac{1}{1+e^{-(1.97-1.48x_{thw}-4.36x_{lvdec3}-2.8x_{lvdec5}+0.85x_{speech})}}$$

Rear-end accident risk model of handheld texting:

(3) $$p=\frac{1}{1+e^{-(1.48-1.28x_{thw}-3.67x_{lvdec3}-2.23x_{lvdec5}+1.27x_{text})}}$$

where $x_{thw}$ is the time headway variable; $x_{lvdec3}$ and $x_{lvdec5}$ are LV deceleration rates of 3 and 5 m/s^2^, respectively; $x_{speech}$ and $x_{text}$ represent speech-based texting and handheld texting, respectively.

According to Equations (2) and (3), the probability curves of rear-end accident can be plotted against the time headway variable (ranging from 0 to 8 s) for non-distracted and distracted driving conditions under LV deceleration rates of 3, 5, and 8 m/s^2^, as shown in Figure 3. Figure 3a compares the rear-end accident probability in conditions of a speech-based texting task with those for no phone use under the different LV decelerations. Similarly, Figure 3b indicates the comparison between the rear-end accident probability in conditions of handheld texting tasks and those of no phone use. As can be seen from Figure 3, the rear-end accident probability decreased with the increase of the time headway, and the accident probability was higher during distracted driving compared with non-distracted driving at the same initial time headway and LV deceleration. For instance, as shown in Figure 3a, the rear-end accident probability of non-distracted driving was 27.1% when the initial time headway was 2 s and the LV deceleration was 8 m/s^2^, while the accident probability increased to 46.5% (approximately two times) if the drivers did not compensate for the increased workload by increasing their time headway during speech-based texting task. However, if the drivers tried to compensate for the increased rear-end accident risk by increasing the time headway, it would be necessary to increase their time headway by 28.5% to maintain the same level of accident risk as was present during the non-distracted driving (i.e., 27.1%). Similarly, as shown in Figure 3b, if a driver was driving at an initial time headway of 2 s and an LV deceleration rate of 8 m/s^2^ during non-distracted driving, the time headway for the handheld texting task needed to be increased by 49.7% to maintain the same accident risk level.

### 3.3. Effectiveness of Driver’s Rear-End Risk Compensation Behavior

**Figure 4:**
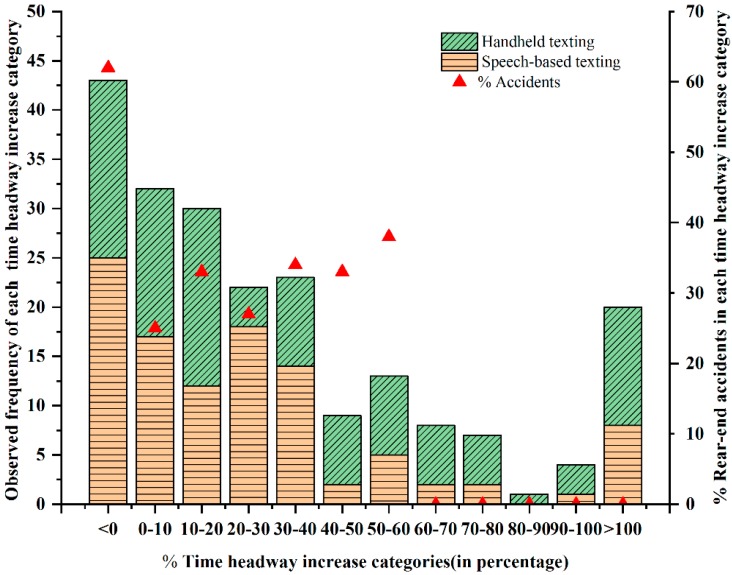
Time headway increase patterns of all drivers during distracted driving conditions.

Section 3.2 presents the required time headway increase estimated from the model for offsetting the increased accident risk associated with mobile phone distracted driving. Whereas, the different drivers perceived different levels of increased accident risk during distracted driving, they also adopted different compensation behaviors by increasing the time headway. In order to gain a deeper understanding of the drivers’ compensation behaviors and to investigate the actual choice of compensation behavior (increasing time headway) of each driver in the distracted driving. The percentage of increased time headway can be obtained in each distracted driving condition compared to non-distracted driving. Figure 4 shows the frequencies of observations of distracted driving conditions and the observed percentage of accident frequency for each time headway increase category. The percentage increase in time headway is classified in 10% intervals (i.e., 0%–10% time headway increase, 10%–20% time headway increase, etc.). Further, the observed distracted driving frequencies in each time headway increase category were plotted by bar charts with horizontal and oblique grid lines (see Figure 4, left Y-axis). The proportion of bar charts with horizontal grid lines in each time headway increase category indicates the number of drivers observed during periods of speech-based texting tasks. Similarly, the proportion of bar charts with oblique grid lines in each time headway increase category demonstrates the number of observations of drivers engaged in handheld texting tasks. For example, the observed distraction driving was 32 in the 0%–10% time headway increase category. A total of 17 drivers were observed during engagement in speech-based texting tasks (the proportion of horizontal grid lines), and the remaining 15 drivers were engaged in handheld texting tasks (the proportion of oblique grid lines). Here, speech-based texting and handheld texting cases include both simple and complex tasks, so there are 106 frequencies in each case. Figure 4 shows that in most of the cases, the driver could increase the time headway up to 40%, and in some of the cases, the drivers could increase the time headway in the range of 40%–80%; however, very few driver’s time headway increase could reach 80%–100%. Interestingly, some drivers increased their time headway by more than 100%; this may be due to the fact that the driver’s risk perception ability was very strong and he or she implemented a larger car-following distance and a lower driving speed to compensate for the increased workload associated with mobile phone use.

To a certain extent, various levels of time headway increase could offset the increased rear-end accident risk; this can be demonstrated as well from the observed percentage of rear-end accident frequency presented by the second Y-axis on the right side of Figure 4. Here, the observed percent accident frequency in each time headway increase category is presented through the red triangle points for both speech-based texting and handheld texting tasks. For example, the rear-end accident probability reached 25% in the 0%–10% time headway increase category. The percentage of accident probability is defined as the ratio of the number of rear-end accidents in each time headway increase category to the number of all the distracted drivers observed in the corresponding time headway increase category. It can also be seen from Figure 4 that the drivers could fully compensate for the increased accident risk (i.e., the rear-end accident probability is 0) when mobile phone distracted drivers increased the time headway to more than 60%. Whereas, when distracted drivers increased the time headway by less than 60%, rear-end accidents caused by mobile phone distraction driving still occurred. 

If the driver did not take risk compensation by increasing the time headway during distracted driving, the results of mobile phone use were worse. As can be seen in Figure 4, among all the categories, the highest rear-end accident probability caused by distracted driving was about 63% (when the distracted driver did not increase time headway to compensate). The data presented in Figure 4 also shows that the driver’s compensation behavior was unsuccessful, because the driver was not able to accurately predict the required compensation amount of time headway to offset the increased accident risk due to the distraction. Therefore, drivers should not use time headway compensation as a strategy to avoid sudden dangerous events. Moreover, too large a time headway increase may also increase the rear-end accident probability from the rear vehicle (because the drivers of the rear vehicle were distracted, or other drivers were taking aggressive action to overtake the slower lead vehicle). 

## 4. Discussion 

This paper examined the impact of mobile phone use (both speech-based texting and handheld texting) on the car-following performance of 53 Chinese drivers in an urban road environment created by a driving simulator. The GEE method was used to develop the time headway model (multiple linear regression) and the rear-end accident risk model (binary logistic regression). The results of the time headway model showed that speech-based texting caused drivers to increase their time headway by 0.41 s, compared with the time headway they chose during periods of non-distracted driving; handheld texting caused drivers to choose a greater increase in time headway (0.59 s). The reason that the drivers chose a larger time headway in conditions of engaging in handheld texting tasks is that handheld texting resulted in greater workload and increased the time they had to take their eyes off the road compared to speech-based texting, and the drivers chose a larger time headway to maintain driving safety [36]. However, the difficulty level of mobile phone distraction tasks had no significant influence on the driver’s choice of time headway. One explanation for this may be that the drivers realized they were being observed, and so to optimize their performance in driving while engaging in secondary tasks, they increased their time headway to a minimum comfort level in both cases. Another explanation is that drivers might not have the same level of engagement in secondary tasks during the complex mobile tasks compared to simple mobile tasks because they wanted to prioritize driving performance; therefore, the driving performance had no significant differences between the two difficulty levels in each task [50]. From the results of the time headway model, it can be clearly seen that the drivers in distracted driving conditions attempted to compensate for the increased accident risk by increasing their time headway to maintain a sufficient level of safe driving [12,23,27].

To study whether a driver’s time headway compensation strategy could completely offset the increased risk of rear-end accidents caused by mobile phone use distraction in car-following situations, the relationship between different time headway increases, different rates of LV deceleration (3, 5, and 8 m/s^2^), and the corresponding rear-end accident probability was analyzed. From this relationship, it can be concluded that rear-end accident probability decreased with the increase in the time headway. The greater the deceleration of the LV, the higher the probability of a rear-end accident. In addition, when the time headway and the LV deceleration were the same, the probability of rear-end accidents in the distracted driving conditions was significantly increased, compared to the non-distracted driving. Furthermore, the results of the rear-end accident risk model also show that the rear-end accident probability decreased by 77% when the time headway increased by 1 s. Compared with the LV deceleration rate of 8m/s^2^, the rear-end accident probability of the LV deceleration rate of 3 m/s^2^ was significantly reduced. Results of the model also indicate that the rear-end accident probability increased by 2.34 and 3.56 times, respectively, during speech-based and handheld texting (compared to non-distraction driving) at LV deceleration rates of 3, 5, and 8 m/s^2^. Interestingly, nevertheless, the effect of the difficulty level of the secondary tasks (i.e., difficulty in speech-based and handheld texting tasks) on the accident probability was not found to be significant. This may be that drivers do not have the same level of involvement during the complex secondary tasks compared to simple secondary tasks because they want to prioritize driving [50]. Compared with speech-based texting tasks, handheld texting had a higher rear-end accident risk. The reason for this may be that the handheld texting task generates more visual distraction, which will cause them to significantly decrease their attention to the road ahead of them [36]. From the actual test data, it may be observed that to completely offset the increased risk of mobile phone distraction in the car-following situation, the expected level of time headway increase should be at least 60%. However, from this study, it can also be observed that about 81% of drivers’ compensation behavior during periods of distracted driving did not reach the required level, indicating that most drivers underestimated the increased rear-end accident risk associated with mobile phone use, especially under different rates of LV deceleration in a car-following situation. 

As the drivers could not accurately assess the risk level of rear-end accidents, they may have chosen the wrong concept of safety compensation during periods of mobile phone distracted driving. Therefore, they did not accurately and effectively compensate for the increased rear-end accident risk caused by mobile phone distraction. Moreover, the current research results also show that drivers should not be encouraged to use mobile phones while driving, even if they adopted the proper time headway compensation strategy during their mobile phone use. 

The present study has certain limitations. Similar to some previous studies, the results of this study were based on a driving simulator experiment; hence, the validity of the results needs further verification by means of natural driving experiments. The participants selected in this study were relatively young and had less driving experience, and the gender distribution was not balanced, with fewer females than males. Future research should increase the sample size to include more middle-aged, elderly, and female drivers in order to generalize the research conclusions to a certain extent. Meanwhile, the research in this paper was carried out in the urban road environment. To further promote the generalization of the research results, other road scenarios (rural roads, highways, and suburban roads) should be simulated and explored in future research.

## 5. Conclusions

Mobile phone use while driving is a common driving behavior and poses a serious threat to public health. To investigate the effects of speech-based texting and handheld texting (each with two difficulty levels) on drivers’ time headway and the effectiveness of the time headway compensation behavior. This study develops the linear regression model for time headway and the binary logistic regression model for accident probability based on the Generalized Estimating Equation method. The results show that drivers perceive an increase in accident risk during the distracted driving, and they adopt risk compensation behavior by increasing time headway to compensate for the increased accident risk associated with mobile phone use. Moreover, the study results also show that the time headway compensation strategy adopted by most drivers cannot completely offset the accident risk associated with mobile phone use. Therefore, the findings of this study can provide theoretical guidance for the formulation of traffic laws related to the use of mobile phones during driving, as well as provide a theoretical basis for driver safety education programs, driver training, and public awareness road safety public awareness campaigns of road safety. Additionally, the rear-end accident risk model established in this study may have practical implications for developing the driver safety warning system and may help to improve driving safety. 

## Figures and Tables

**Table 1:** Descriptive statistics of the participants obtained from the questionnaire.

Variables	Description	Type	Levels	Mean	SD	Percentage
Driver demographics						
Age	–	Con	–	25.25	3.08	–
Gender	–	Cat	2	–	–	–
Male	1	–	–	–	–	69.81
Female *	2	–	–	–	–	30.19
Driving history						
Years of driving	–	Con	–	3.02	2.27	–
Driven kilometers	–	Cat	3	–	–	–
0–5000 km *	1	–	–	–	–	79.25
5000–10,000 km	2	–	–	–	–	9.43
>10,000 km	3	–	–	–	–	11.32
Crash involvement history in the last three years	–	Cat	3	–	–	–
None *	1	–	–	–	–	96.23
Once	2	–	–	–	–	1.89
More than once	3	–	–	–	–	1.89
Traffic accidents due to mobile phone use	–	Cat	3	–	–	–
None *	1	–	–	–	–	100
Once	2	–	–	–	–	0
More than once	3	–	–	–	–	0
Mobile phone use habits						
Frequency of speech-based texting use while driving	–	Cat	3	–	–	–
None or less *	1	–	–	–	–	39.62
Sometimes	2	–	–	–	–	41.51
Frequently	3	–	–	–	–	18.87
Frequency of handheld texting use while driving	–	Cat	3	–	–	–
None or less *	1	–	–	–	–	64.15
Sometimes	2	–	–	–	–	33.96
Frequently	3	–	–	–	–	1.89

Note. Cat = categorical variable, Con = continuous variable; SD = Standard Deviation; * Reference category.

**Table 2:** Results of the Generalized Estimating Equation (GEE) models for time headway at lead vehicle (LV) brake onset in different driving conditions.

Speech-Based Texting					Handheld Texting			
Parameter	Estimate	SE	z	*p* > |z|	Estimate	SE	z	*p* > |z|
Intercept	1.97	0.16	12.67	<0.001	2.17	0.16	13.91	<0.001
Gender (Female *)	0.07	0.19	0.38	0.702	−0.05	0.19	−0.24	0.808
Speech-based texting (No phone *)	0.41	0.10	4.20	<0.001				
Difficulty in speech-based texting (Simple *)	0.15	0.11	1.37	0.169				
Handheld texting (No phone *)					0.59	0.09	6.37	<0.001
Difficulty in handheld texting (Simple *)					0.01	0.11	0.14	0.890
The goodness of fit for models	Wald chi2(3) = 19.64, Prob > chi2 = 0.0002				Wald chi2(3) = 40.71, Prob > chi2 = 0.0000			

SE = Standard Error; * Reference category.

**Table 3:** Model results for rear-end accident risk across different driving conditions.

Speech-Based Texting						Handheld Texting				
Parameter	Estimate	SE	z	*p* > |z|	OR	Estimate	SE	z	*p* > |z|	OR
Intercept	1.97	0.67	2.96	0.003	7.19	1.48	0.63	2.37	0.018	4.41
Gender (Female *)	−0.58	0.42	−1.37	0.170	0.56	−0.02	0.38	−0.05	0.964	0.98
Initial time headway	−1.48	0.36	−4.11	<0.001	0.23	−1.28	0.29	−4.44	<0.001	0.28
Lead vehicle deceleration 3 (Lead vehicle deceleration 8 m/s^2^ *)	−4.36	1.05	−4.16	<0.001	0.01	−3.67	0.75	−4.89	<0.001	0.03
Lead vehicle deceleration 5 (Lead vehicle deceleration 8 m/s^2^ *)	−2.81	0.56	−5.04	<0.001	0.06	−2.23	0.45	−4.98	<0.001	0.11
Speech-based texting (No phone *)	0.85	0.40	2.11	0.035	2.34					
Difficulty in speech-based texting (Simple *)	−0.04	0.43	−0.10	0.922	0.96					
Handheld texting (No phone *)						1.27	0.40	3.15	0.002	3.56
Difficulty in handheld texting (Simple *)						0.30	0.40	0.75	0.453	1.35
The goodness of fit for models	Wald chi2(6) = 50.03, Prob > chi2 = 0.0000					Wald chi2(6) = 58.11, Prob > chi2 = 0.0000				

SE = Standard Error; OR: Odds Ratio; * Reference category.
